# Synthesis, Characterization and Structural Elucidation of Biocompatible Hydroxychloroquine–Zinc Oxide Hybrid Nanoclusters as Synergistic Antiplasmodial Agents

**DOI:** 10.3390/pharmaceutics18101221

**Published:** 2026-09-26

**Authors:** Swati Rani, Balvinder Kumar, Jai Devi, Anju Manuja

**Affiliations:** 1 ICAR-National Research Centre on Equines, Sirsa Road, Hisar 125001, Haryana, India; spahwa999@gmail.com (S.R.); bmanuja.nrce@gmail.com (B.K.); 2 Department of Chemistry, Guru Jambheshwar University of Science and Technology, Hisar 125001, Haryana, India

**Keywords:** hydroxychloroquine, zinc oxide nanoclusters, antimalarial activity, plasmodium falciparum, molecular docking, drug delivery, anti-inflammatory activity, nanomedicine

## Abstract

**Background:** The increasing emergence of antimalarial drug resistance and safety concerns associated with conventional therapies necessitates the development of improved compounds. In this study, we report the synthesis of hydroxychloroquine–zinc oxide hybrid nanoclusters (HCQ-ZnONPs) by integrating a bioactive organic ligand (hydroxychloroquine) with a bioactive metal oxide nanocluster (ZnONPs). **Methods:** Structural and physicochemical characterization was performed using proton nuclear magnetic resonance (^1^H NMR), fourier-transform infrared spectroscopy (FT-IR), mass spectrometry, X-ray diffraction (XRD), transmission electron microscopy (TEM), field emission scanning electron microscopy (FE-SEM), energy-dispersive X-ray analysis (EDAX), dynamic light scattering (DLS), and zeta potential. Molecular docking against *Plasmodium falciparum* dihydrofolate reductase–thymidylate synthase (PfDHFR-TS) was performed to investigate potential ligand–target interactions. Drug loading and HCQ release were evaluated at pH 7.4. We also assessed in vitro cytocompatibility, nitric oxide estimation, total antioxidant capacity, anti-inflammatory and antimalarial activities. **Results:** The physicochemical characterization supported the successful formation of HCQ-ZnONPs. In the proposed model of HCQ-ZnONPs, a quinoline nitrogen of the HCQ molecule interacts with an exposed Zn^2+^ site on the surface of a ZnONPs cluster. The HCQ-ZnONPs exhibited a drug loading of 21.4% and showed approximately 48.4% cumulative HCQ release over 24 h at pH 7.4. To the best of our knowledge, this is the first report of an HCQ-ZnONPs nanocluster system. Biological evaluation demonstrated synergistic in vitro antimalarial activity, indicating enhanced efficacy compared with the parent drug, suggesting that this arises from the integration of HCQ and ZnO nanoclusters while maintaining favorable cytocompatibility in Vero cells. The oxidative stress and anti-inflammatory potential of the hybrid complex was also assessed, highlighting its multifunctional biological profile that may be advantageous for mitigating malaria-associated oxidative stress and inflammatory responses. Molecular docking against PfDHFR-TSfurther supported favorable ligand-target interactions and provided additional insight into the potential molecular basis of the observed biological activity. **Conclusions:** Collectively, the experimental, theoretical, and mathematical analysis suggests the successful formation of HCQ-ZnONPs with promising anti-inflammatory and antimalarial efficacy, establishing this hybrid nano system as a potential candidate for next-generation antiplasmodial therapy.

## 1. Introduction

Hydroxychloroquine (HCQ), a 4-aminoquinoline analog of chloroquine, has long been recognized for its broad pharmacological utility, extending from antimalarial action to immunomodulatory and anti-inflammatory applications [1,2,3]. Originally developed as a safer alternative to chloroquine, HCQ has become widely used in the management of autoimmune disorders such as rheumatoid arthritis and systemic lupus erythematosus due to its comparatively improved safety profile and immunoregulatory properties [4,5]. Its pharmacological activity is closely associated with its ability to elevate lysosomal and endosomal pH, thereby modulating autophagy, antigen processing, and toll-like receptor (TLR)-mediated signaling pathways. Consequently, the secretion of pro-inflammatory mediators including IL-1, IL-6, and TNF-α is significantly reduced, accounting for its biological relevance across inflammatory and infectious conditions [6,7]. In malaria, HCQ and related quinoline derivatives interfere with heme detoxification within Plasmodium parasites, resulting in toxic heme accumulation and subsequent parasite death. However, prolonged administration of HCQ is associated with adverse effects such as retinopathy, cardiomyopathy, and myopathy, which limit its long-term application [8,9]. Therefore, the development of nano-enabled HCQ systems capable of improving biological performance while reducing associated toxicity has gained increasing attention.

Nanotechnology-based compounds have emerged as promising approaches to enhance the pharmacological performance of conventional antimalarial compounds. Several studies have reported hydroxychloroquine or chloroquine complexes with transition metal ions, including Zn(II), Cu(II), Au(I), Pt(II), and Ru(II), in which metal ions derived from soluble metal salts coordinate with the drug molecule to modify its physicochemical or biological properties [10,11,12]. Owing to their nanoscale dimensions and high surface-area-to-volume ratio, a single nanoparticle contains several exposed metal atoms, allowing multiple ligands to coordinate or bind simultaneously; thus, enhanced interaction capabilities facilitate efficient drug delivery [13,14]. Unlike classical coordination complexes, metal oxide nanoparticles, including ZnONPs, provide a nanoscale surface that can interact with drugs [15]. Among various nanomaterials, zinc oxide nanoparticles (ZnONPs) have attracted considerable interest owing to their intrinsic antimicrobial, antioxidant, anti-inflammatory, and biocompatible properties [16,17]. In addition, zinc plays an important role in immune regulation, enzymatic activity, and inhibition of microbial growth. Interestingly, HCQ has been reported to function as a zinc ionophore, promoting intracellular zinc transport and accumulation, thereby providing a mechanistic basis for combining HCQ with ZnONPs to achieve synergistic biological effects [14,18]. Furthermore, ZnONPs may enhance membrane permeability and cellular uptake, potentially improving antiplasmodial activity while simultaneously contributing antioxidant and anti-inflammatory properties that may help alleviate malaria-associated oxidative stress and inflammatory responses. Malaria remains one of the most significant parasitic diseases worldwide, with the emergence of resistance to conventional antimalarial drugs posing a major global health concern. The erythrocytic stage of *Plasmodium* infection is associated with excessive oxidative stress, inflammatory cytokine production, and red blood cell membrane damage, all of which contribute substantially to disease progression. Hence, the development of multifunctional systems capable of simultaneously exhibiting antiplasmodial, antioxidant, anti-inflammatory, and hemocompatible properties represents an important area of current research. To the best of our knowledge, no previous study has comprehensively investigated hydroxychloroquine-associated ZnONPs for antiplasmodial applications.

In the present study, we report the synthesis and characterization of an HCQ-ZnONPs nanoformulation integrating a pharmacologically active organic ligand with a bioactive metal oxide nanocluster. Structural and physicochemical characterization using ^1^H NMR, FT-IR, mass spectrometry, XRD, SEM, TEM, DLS-Zeta, and EDAX analyses supports the association of the HCQ-ZnONPs complex. The biological performance of the synthesized nanoformulation was evaluated through in vitro antimalarial screening using a modified Rieckmann micro-assay, cytotoxicity assessment in Vero cells using the resazurin assay, anti-inflammatory activity using the BSA denaturation model, nitric oxide scavenging activity, and total antioxidant capacity evaluation. In addition, molecular docking studies against *Plasmodium falciparum* dihydrofolate reductase-thymidylate synthase (PfDHFR-TS) were performed to investigate possible molecular interactions associated with antiplasmodial and anti-inflammatory activity. Collectively, the findings indicate that the HCQ-ZnONPs nanoformulation exhibits notable antiplasmodial activity, favorable biocompatibility, and multifunctional biological properties, highlighting its relevance as a promising nano-enabled antimalarial system for further investigation.

## 2. Experimental

### 2.1. Materials & Synthesis

The chemicals used in the experimental work were of analytical reagent (AR) grade and included zinc(II) sulfate heptahydrate (≥99%), sodium hydroxide pellets (≥99%), diethyl ether (≥99%), and methanol (≥99%), all sourced from Sigma-Aldrich Chemicals Private Limited, Bengaluru, India.. Hydroxychloroquine sulfate (≥99%) from tablet, HCQS-400, IPCA Pharma, IPCA Laboratories Ltd., Mumbai, India. The glassware employed in the synthesis process was meticulously cleaned with acids and acetone. No further purification of the chemicals was performed before use.

Zinc oxide nanoparticles (ZnONPs) were synthesized using zinc sulfate heptahydrate as the zinc precursor and sodium hydroxide as the precipitating agent through a modified microwave-assisted pulsed heating method reported previously. The resulting precipitate was collected by centrifugation, repeatedly washed with distilled water, and dried in a microwave oven operating at 600 W using alternating heating cycles of 2 min on and 3 min off [19,20].

For complexation, the synthesized ZnONPs were mixed with hydroxychloroquine (HCQ) in methanol at a ZnONPs:HCQ molar ratio of 1:2. The reaction mixture was refluxed at 80 °C under continuous stirring (678 rpm) for 3–4 h and subsequently stirred overnight at room temperature to ensure complete association. The resulting solid product was isolated by filtration, washed successively with methanol and diethyl ether, and recrystallized to obtain the purified compound [21].

### 2.2. Molecular Docking

#### 2.2.1. Molecular Docking and Computational Analysis

The molecular docking studies were performed to investigate the interaction of hydroxychloroquine (HCQ) and the synthesized HCQ-ZnONPs with *Plasmodium falciparum* dihydrofolate reductase-thymidylate synthase (PfDHFR-TS). The crystal structure of PfDHFR-TS was obtained from the Protein Data Bank using PDB ID: 3QGT. The crystal structure contains the enzyme in complex with the co-crystallized inhibitor CP6 and was selected to define the experimentally observed inhibitor-binding region [22,23].

#### 2.2.2. Protein and Binding-Site Preparation

The 3QGT protein structure was prepared using BIOVIA Discovery Studio 2022. The co-crystallized ligand CP6 was used as a reference to identify the inhibitor-binding pocket. The receptor binding site was defined from the position of CP6, and a spherical binding region was generated around the crystallographic ligand. The resulting binding-site sphere, with coordinates 28.0348, 5.43635, 58.756 Å and a radius of 6.37018 Å, was used consistently for docking of both HCQ and the HCQ-ZnONPs model. Following definition of the binding site, the crystallographic inhibitor was removed from the receptor structure before docking. The prepared receptor was subsequently used for the docking calculations.

#### 2.2.3. Preparation of HCQ and HCQ-ZnONPs

The two-dimensional chemical structure of HCQ was converted into a three-dimensional structure using BIOVIA Discovery Studio. HCQ ligand preparation was performed using the Prepare Ligands protocol. Possible ionization states were generated within a pH range of 6.5–8.5, and possible tautomeric and stereochemical forms were generated. Bad valences were corrected where necessary, and three-dimensional coordinates were generated for the resulting ligand structures.

The HCQ-ZnONPs model was constructed based on the experimentally predicted association of four molecules of ZnO with one HCQ through the quinolone nitrogen. HCQ-ZnONPs were converted into a three-dimensional molecular representation and saved in SDF format for subsequent computational analysis.

#### 2.2.4. Receptor–Ligand Interaction Analysis 

Docking calculations were performed using the CDOCKER protocol implemented in BIOVIA Discovery Studio. HCQ and the HCQ-ZnONPs models were evaluated against the same CP6-defined binding site of PfDHFR-TS to allow comparison of their predicted binding orientations within the enzyme pocket.

The selected docking poses were subjected to receptor–ligand interaction analysis using BIOVIA Discovery Studio. The interactions between the docked molecules and amino acid residues within the PfDHFR-TS binding region were examined, including hydrogen bonds, hydrophobic interactions, electrostatic interactions, and aromatic/π interactions where applicable.

### 2.3. Characterization

The synthesized compounds were characterized using complementary spectroscopic and microscopic techniques to determine their structural, morphological, elemental, and physicochemical properties.

#### 2.3.1. High-Resolution Transmission Electron Microscopy (HR-TEM)

HR-TEM analysis was carried out using a JEOL transmission electron microscope, Ltd., Akishima, Tokyo, Japan. The samples were dispersed in distilled water, ultrasonicated to obtain homogeneous suspensions, deposited on carbon-coated copper grids, and air-dried prior to imaging. Particle morphology, crystallinity, and size distribution were subsequently evaluated.

#### 2.3.2. Dynamic Light Scattering and Zeta Potential

Hydrodynamic particle diameter, polydispersity index (PDI), and surface charge of the compounds were measured using a Litesizer 500 particle analyzer, Anton Paar GmbH, Graz, Styria, Austria. Prior to analysis, the samples were sonicated in distilled water to obtain uniform dispersions. Dynamic light scattering and electrophoretic light scattering modes were used for particle size and zeta potential measurements, respectively.

#### 2.3.3. Proton Nuclear Magnetic Resonance (^1^H NMR)

The ^1^H NMR spectra of hydroxychloroquine and the associated compound were recorded in deuterium oxide (D_2_O) using a Bruker Avance III 400 MHz spectrometer, Bruker Corporation, Billerica, MA, USA. Sodium 3-(trimethylsilyl)propane sulfonate (TSP) was employed as the internal chemical shift reference.

#### 2.3.4. Fourier Transform Infrared Spectroscopy (FT-IR)

Infrared spectra were acquired using a PerkinElmer BX II FT-IR spectrometer, PerkinElmer, Waltham, MA, USA, with KBr pellet methodology over the spectral range of 4000–400 cm^−1^ to identify characteristic functional groups and confirm coordination.

#### 2.3.5. Mass Spectrometry

Mass spectral analysis was performed on a SCIEX Triple TOF 5600 instrument, SCIEX, Concord, ON, Canada, using acetonitrile as the solvent. The obtained mass-to-charge (*m*/*z*) values were used to verify the molecular composition of the synthesized compounds.

#### 2.3.6. Powder X-Ray Diffraction (XRD)

Powder X-ray diffraction patterns were collected using a Rigaku Miniflex-II diffractometer, Rigaku Corporation, Akishima, Tokyo, Japan, equipped with Cu Kα radiation (λ = 1.5406 Å). Crystallinity and average crystallite size were evaluated using the Debye–Scherrer equation:

D = kλ/βcos θ

where:

- D = crystallite size (in nanometers);
- k = Scherrer constant (typically 0.9);
- λ = wavelength of the X-ray radiation (for Cu Kα, λ = 1.5406 Å);
- β = full width at half maximum (FWHM) of the diffraction peak (in radians);
- θ = Bragg angle.

#### 2.3.7. Field Emission Scanning Electron Microscopy (FE-SEM)

Surface morphology of the synthesized samples was examined using a JEOL JSM-7610F Plus scanning electron microscope, JEOL Ltd., Akishima, Tokyo, Japan after gold sputter coating. Elemental composition and elemental distribution were determined using energy-dispersive X-ray spectroscopy (EDX) and elemental mapping.

#### 2.3.8. Molar Conductivity and Melting Point

Molar conductivity was measured using an EUTECH PC2700 conductivity meter, Thermo Fisher Scientific, Waltham, MA, USA. Melting point determination was performed by the open capillary method using a Gallenkamp melting point apparatus.

### 2.4. Biological Evaluation

#### 2.4.1. Cytotoxicity Assay

The cytotoxic potential of synthesized compounds was assessed against Vero cells using the resazurin reduction assay. Cells were cultured in complete growth medium containing fetal bovine serum, HEPES buffer, L-glutamine, sodium bicarbonate, penicillin, streptomycin, and amphotericin B before seeding into 96-well plates. Following incubation to approximately 70% confluence, cells were treated with different concentrations (10–1.25 mg mL^−1^) of the synthesized compounds for 24 h. Resazurin solution (1 mg mL^−1^) was then added and incubated for an additional 4 h. Fluorescence resulting from conversion of resazurin to resorufin was quantified at 590 nm, and cell viability was expressed relative to untreated controls [24].

#### 2.4.2. Nitric Oxide Estimation

Nitric oxide production was quantified using the Griess colorimetric method. Equal volumes of culture supernatant and freshly prepared Griess reagent were mixed and incubated at 37 °C for 10 min. Absorbance was recorded at 540 nm, and nitric oxide concentration was calculated using a sodium nitrite calibration curve [25,26].

#### 2.4.3. Total Antioxidant Capacity

Total antioxidant capacity was determined using a Total Antioxidant Capacity (T-AOC) Colorimetric Assay Kit (E-BC-K136-M), Elabscience Biotechnology Inc., Houston, TX, USA. Cell culture supernatants obtained after treatment with the synthesized compounds were incubated with cupric ion reagent according to the manufacturer’s protocol. Following incubation for 90 min in the dark, absorbance was measured at 570 nm. Antioxidant activity was calculated from a Trolox standard curve and expressed as Trolox equivalents [27].

#### 2.4.4. Anti-Inflammatory Activity

Anti-inflammatory activity was evaluated using the bovine serum albumin (BSA) protein denaturation assay. Stock solutions (1 mg mL^−1^) were prepared in DMSO and serially diluted with phosphate buffer (0.2 M, pH 7.4). The test solutions were mixed with BSA solution and incubated at 37 °C before heat-induced denaturation at 70 °C. After cooling, absorbance was recorded at 660 nm. The percentage inhibition of protein denaturation was calculated and plotted as percent inhibition [28,29,30,31].

#### 2.4.5. In Vitro Antimalarial Activity

The antimalarial activity of the synthesized compounds was evaluated against *Plasmodium falciparum* (3D7 strain) using a modified Rieckmann microtest. Parasites were cultured in RPMI-1640 medium supplemented with HEPES, glucose, sodium bicarbonate, and heat-inactivated human serum. Ring-stage parasites synchronized with 5% D-sorbitol were adjusted to 0.8–1.5% parasitaemia at 3% hematocrit. Serially diluted compounds were added to parasite cultures and incubated at 37 °C in a candle jar for 36–40 h. Thin blood smears prepared after incubation were stained using Jaswant Singh Bhattacharya (JSB) stain, and inhibition of schizont maturation was examined microscopically. The minimum inhibitory concentration was recorded as the lowest concentration preventing schizont development [32,33].

### 2.5. Drug Loading and Encapsulation Efficiency

The amount of HCQ in the complex was calculated by the difference between the total amount of drug added and the amount of unbound drug remaining in the aqueous supernatant at the time of preparation. The amount of HCQ loaded in 25 mg of ZnONPs was also calculated and was represented as wt%. The supernatant was collected, measured and analyzed for free drug content spectrophotometrically at 343 nm. A calibration curve of the drug was prepared using a stock solution (10 mg/mL) in distilled water. In order to obtain a calibration graph, a standard solution of the drug was twofold serially diluted with distilled water to obtain solutions with concentrations ranging from 2.5 to 0.3125 mg/mL. The UV absorption intensity was measured at 343 nm against distilled water as a blank. Encapsulation efficiency and drug loading were then calculated using Equations (1) and (2):

(1) (%) Encapsulation efficiency (EE %) = W_t_/W_i_ × 100

(2) Drug loading (%) = W_t_/W_n_ × 100

where W_t_ is the total amount of drug in the complex [Total HCQ − free HCQ}] W_i_ is the total quantity of HCQ added initially during preparation and W_n_ is the total weight of drug-loaded nanoparticles [34].

### 2.6. In Vitro Drug Release Study

The in vitro release of HCQ from the synthesized HCQ-ZnONPs complex was evaluated using a dialysis membrane. Briefly, 30 mg of the HCQ-ZnONPs was dispersed in 3 mL (10 mg/mL) of phosphate-buffered saline (PBS) as the release medium and transferred into a pre-treated dialysis membrane. The sealed dialysis bag was immersed in 100 mL of PBS (pH 7.4) maintained at 37 ± 0.5 °C under continuous magnetic stirring at approximately 120 rpm. The release experiment was performed for 24 h.

At predetermined time intervals (0, 0.5, 1, 2, 4, 6, 12, and 24 h), 1 mL of the external release medium was withdrawn for analysis. After each sampling, an equal volume (1 mL) of fresh, pre-warmed PBS was added to maintain a constant release-medium volume of 100 mL. The collected samples were analyzed for HCQ content using a microplate/ELISA reader at 343 nm. A calibration curve of the drug was prepared by using a stock solution (10 mg/mL), two-fold diluted to obtain solutions with concentrations ranging from 250 to 1.95 µg/mL in distilled water [34].

The cumulative amount of HCQ released was calculated by taking into account the volume of release medium removed at each sampling interval and subsequently replaced with fresh PBS. The cumulative amount of HCQ released at each time point was calculated according to:

(3) M_t_ = C_t_ V + V_s_ ∑C_previous_

where M_t_ is the cumulative amount of HCQ released at time t, C_t_ is the HCQ concentration in the release medium at time t, V is the total volume of release medium (100 mL), V_s_ is the volume of sample withdrawn at each time point (1 mL), and represents the HCQ concentrations measured at the preceding sampling intervals and ∑C_previous_ is the sum of concentrations measured at all previous sampling points.

The cumulative percentage of HCQ released was calculated using:

Cumulative HCQ release (%) = (M_t_/M_total HCQ_) × 100

where M_total HCQ_ represents the total amount of HCQ present in the 30 mg HCQ-ZnONPs sample, calculated from the experimentally determined drug-loading percentage.

The release profile was expressed as cumulative HCQ release (%) versus time (h). All measurements were performed in duplicate.

To analyze release kinetics and mechanism, data obtained for in vitro release were fitted into mathematical equations [35,36,37,38]

- Cumulative percentage of release versus time (zero order);
- Cumulative percentage of drug retained versus time (first order);
- Cumulative percentage of release versus root ‘T’ (Higuchi’s classical diffusion equation);
- Log of cumulative percentage of drug released versus log time (Korsmeyer–Peppas).

The data were fitted to the exponential equation (Korsmeyer–Peppas equation) to confirm the best-fit model:

(4) M_t_/M_∞_ = kt^n^

where M_t_ is the amount of drug released at time t, M_∞_ is the total amount of drug present in the formulation, k is the release rate constant and depends on the geometry of the complex, and n is the diffusion exponent indicating the mechanism of drug release.

### 2.7. Statistical Analysis

Experimental data were analyzed using GraphPad Prism version 10.0. Statistical significance was determined by two-way analysis of variance (ANOVA) to evaluate the effects of treatment, concentration, and their interaction. Differences were considered statistically significant at *p* < 0.05.

## 3. Results and Discussion

### 3.1. Synthesis

The synthesis of ZnONPs was carried out using zinc(II) sulfate heptahydrate as the precursor and sodium hydroxide as the reducing agent. Furthermore, HCQ-ZnONPs were synthesized by combining HCQ with pre-synthesized ZnONPs (Figure 1). Due to the high surface-area-to-volume ratio of ZnONPs, it contains several exposed metal atoms, allowing multiple ligands to coordinate or bind simultaneously. Theoretically, HCQ may interact with the nanoparticle surface through several possible mechanisms, including monodentate surface coordination, surface adsorption through multiple weak interactions, bridging between adjacent surface Zn^2+^ sites, and electrostatic interactions involving the hydroxyl and amino groups [39,40,41]. The structural features and intermolecular interactions of both ZnONPs and the HCQ-ZnONPs were systematically investigated using various physicochemical and spectroscopic techniques, including FT-IR, ^1^H NMR, and mass spectrometry (Table 1). Previous investigations were limited to computational studies suggesting the potential use of (ZnO)n nanoparticles for hydroxychloroquine delivery [42,43].

**Figure 1:**
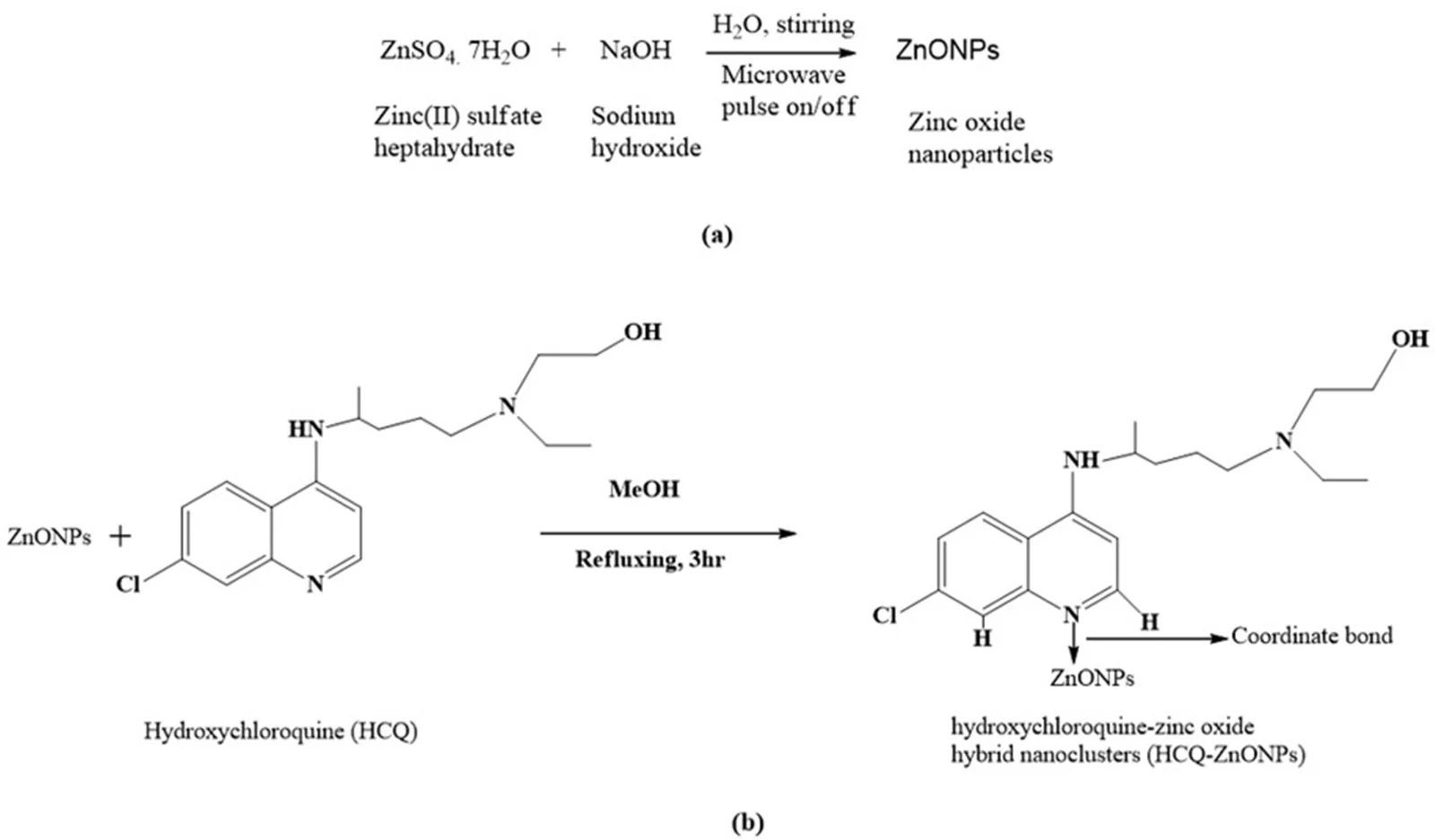
Proposed structure of ZnONPs (**a**) and HCQ-ZnONPs (**b**).

**Table 1:** Physical properties of synthesized compounds HCQ, ZnONPs, and HCQ-ZnONPs.

Sr. No.	Compound	Molecular Formula	Color	*m*/*z*	Theoretical Molecular Mass	Molar Conductivity (μs)	Melting Point (°C)
1	HCQ	C_18_H_25_ClN_3_OH	White	432.2989	433.95	86.47	210
2	ZnONPs	(ZnO)_4_	White	325.2360	325.64	64.42	1975
3	HCO-ZnONPs	C_18_H_26_OClN_3_Zn_4_O_4_	White	759.2528	759.59	160.8	230

### 3.2. Molecular Docking

Molecular docking analysis was performed using the CDOCKER protocol in BIOVIA Discovery Studio to investigate the predicted binding modes of HCQ and HCQ-ZnONPs against *Plasmodium falciparum* dihydrofolate reductase–thymidylate synthase (PfDHFR-TS; PDB ID: 3QGT). Under the same docking conditions, HCQ showed a CDOCKER Energy of 29.7219 and a CDOCKER Interaction Energy of 48.8042 (Table 2), whereas HCQ-ZnONPs exhibited corresponding values of 41.4072 and 72.3525, respectively. Thus, HCQ-ZnONPs produced higher CDOCKER scores than HCQ under the applied scoring protocol, including a higher predicted receptor–ligand interaction energy. The interaction analysis of HCQ indicated contacts with residues including Asp54, Phe58, Met55, Ile112, and Phe116, involving hydrogen-bonding, electrostatic, and hydrophobic/π-associated interactions (Figure 2a). In comparison, the HCQ-ZnONPs model showed interactions involving Asp54, Phe58, Met55, Ile112, Ile14, Leu46, Lys49, Pro113, and the region surrounding the cofactor NDP (A:610). The Zn-containing portion of the model also showed predicted electrostatic/metal-associated contacts within the binding region, particularly around Asp54 and Phe58 (Figure 2b). These findings indicate that incorporation of the ZnO-containing component altered the predicted interaction pattern and produced higher CDOCKER scores under the same computational conditions. The docking results are therefore considered supportive and hypothesis-generating, requiring experimental validation.

**Table 2:** Molecular docking results of HCQ and HCQ-ZnONPs in the binding pocket of 3QGT.

Sr. No.	Compound	-CDOCKER Energy	-CDOCKER Interaction Energy	Key Interacting Residues
1	HCQ	29.7219	48.8042	Asp54, Phe58, Met55, Ile112, and Phe116
2	HCQ-ZnONPs	41.4072	72.3525	Asp54, Phe58, Met55, Ile112, Ile14, Leu46, Lys 49, Pro113, Ile112, Met55, Phe58, Ndp610

**Figure 2:**
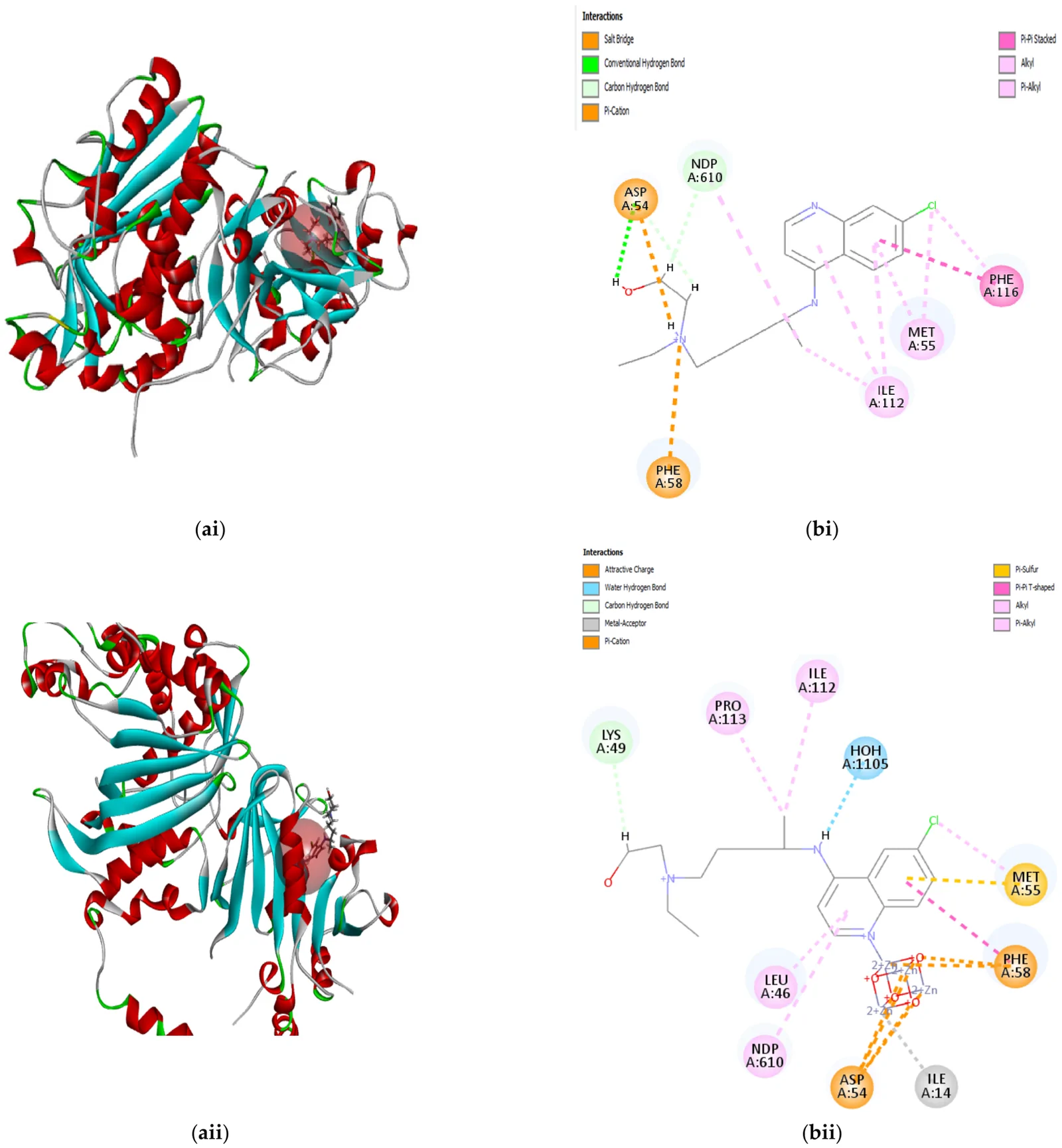
(**a**) 3D interactions; (**b**) 2D diagram: (**i**) HCQ; (**ii**) HCQ-ZnONPs with PfDHFR-TS protein.

### 3.3. Particle Size, Morphology and Surface Characteristics

Transmission electron microscopy (TEM) analysis revealed that both pristine ZnONPs and HCQ-ZnONPs consisted predominantly of spherical/quasi-spherical nanosized particles (Figure 3). The particles of ZnONPs exhibited nanoscale dimensions with an approximate width of 25 nm, while in the complex, the spherical assemblies showed an estimated size of 15 nm. Compared with pristine ZnONPs, the HCQ-ZnONPs exhibited a greater degree of particle aggregation, together with the presence of low-contrast amorphous regions surrounding the nanoparticle clusters, consistent with the association of HCQ on the ZnONPs surface. The crystalline nanoparticle morphology remained preserved after HCQ association.

Dynamic light scattering (DLS) analysis showed an average hydrodynamic diameter of 853 nm, indicating the presence of nanoparticle aggregates in aqueous suspension. DLS does not measure the literal, physical dry core of a particle. Instead, it measures the hydrodynamic diameter, which arises from light scattering and inherently includes the particle’s core plus the surrounding shell of solvent molecules (the solvation layer); when particles aggregate, this results in a larger value. Such differences between TEM and DLS measurements are commonly reported for metal oxide nanoparticle dispersions.

The electrostability of the HCQ-ZnONPs was further evaluated by zeta potential analysis. The complex exhibited a zeta potential of −16.3 mV, improved from ZnONPs with −10.3 mV reported previously [44]. The observed surface charge is consistent with the aggregation behavior observed in the TEM and DLS analyses.

Collectively, the TEM, DLS, and zeta potential results may indicate that HCQ association did not alter the fundamental nanoscale morphology of the ZnONPs but influenced their aggregation behavior in suspension. These findings, together with the complementary spectroscopic and structural analyses, support the successful preparation of the HCQ-ZnONPs for subsequent in vitro biological evaluation.

### 3.4. ^1^H NMR Spectra

In the ^1^H NMR spectrum of HCQ (Figure 4a), characteristic signals were observed in both aliphatic and aromatic regions. In the aliphatic region, a triplet at δ 1.12–1.15 ppm corresponds to the methyl protons of the terminal diethylamino group, while a multiplet at δ 2.95–3.10 ppm is attributed to the methylene protons adjacent to the nitrogen atom. A singlet at δ 4.00 ppm corresponds to the benzylic proton attached to the quinoline ring, and a signal at δ 5.87 ppm is assigned to the hydroxyl proton of the hydroxyethyl chain.

Following complexation with ZnONPs (Figure 4b), the aliphatic region exhibited only minor chemical shift variations, suggesting that the terminal diethylamino group remains uninvolved in coordination. However, a notable upfield shift in the benzylic proton to δ 3.25 ppm was observed, indicating a change in its electronic environment due to metal-ligand interaction.

In the aromatic region, the peaks of HCQ at δ 6.65–6.68, 7.30–7.36, and 7.55 ppm shifted downfield to δ 7.48–7.50, 7.70–7.72, and 8.05 ppm in the HCQ-ZnONPs complex. These downfield shifts may be attributed to deshielding induced by coordination between the quinoline nitrogen atom and Zn^2+^ sites on the nanoparticle surface.

### 3.5. Mass Spectral Analysis

The mass spectral analysis of HCQ (Figure 5a), ZnONPs (Figure 5b), and HCQ-ZnONPs (Figure 5c) was performed in acetonitrile using positive ion mode, which reliably provided evidence for the synthesis and stoichiometry by correlating the *m*/*z* values with the molecular masses. HCQ exhibited its characteristic molecular ion peak at *m*/*z* 432.2989, whereas ZnONPs showed cluster-related ions centered around *m*/*z* 325.2360, consistent with the formation of a small ZnO cluster.

The mass spectrum of the HCQ-ZnONPs displayed several ions at *m*/*z* 536.1873, 610.2095, 684.2312, 759.2542, 832.2747, and 906.2970, indicating the presence of HCQ-ZnONPs species. Among these, the ion at *m*/*z* 759.2542 suggested the calculated mass of an HCQ-ZnONPs species containing four ZnO units and was therefore selected for structural interpretation. The literature also supports the formation of four ZnO unit’s cluster [45]. Therefore, we propose a model of HCQ-ZnONPs, where a quinoline nitrogen of the HCQ molecule interacts with an exposed Zn^2+^ site on the surface of a ZnONPs cluster. The remaining ions are attributed to additional associated ZnO species and ionization-derived cluster/adduct ions, which are commonly observed in the mass spectra of nanoparticle-based systems.

### 3.6. XRD Analysis

The amorphous or crystalline nature of the compound was determined by powder XRD analysis at ambient temperature over the 2θ range of 10–80°. The XRD pattern of ZnONPs synthesized under identical conditions shows sharp diffraction peaks indexed to the hexagonal wurtzite phase (JCPDS No. 36-1451) [46]. In contrast, the HCQ-ZnONPs complex exhibits a broad diffraction without distinct Bragg reflections, indicating an amorphous or poorly crystalline material. This suggests that hydroxychloroquine acts as a coordinating ligand, interrupting Zn–O condensation during nanoparticle growth and may stabilize small Zn-O aggregates rather than allowing the formation of extended crystalline wurtzite ZnONPs (Figure 6).

### 3.7. SEM Analysis

The SEM images revealed the surface morphology of ZnONPs, and it was found that the it exhibits a rod-like shape. In contrast, the HCQ-ZnONPs showed a flattened leaf-like morphology, indicating a significant morphological transformation after association. Elemental identification and quantitative compositional analysis were performed using energy-dispersive X-ray spectroscopy (EDAX) (Figure 7). The EDAX spectra for ZnONPs indicate the presence of the expected elements, zinc (Zn) and oxygen (O), with weight percentages of Zn (55.4%) and O (44.6%), which are in good agreement with the stoichiometric composition of ZnO and indicate high purity of the synthesized nanoparticles.

For the complex, the EDAX results showed the presence of carbon (C), oxygen (O), nitrogen (N), and zinc (Zn), showing association. The quantitative analysis revealed weight percentages of C (34.3%), O (54.3%), N (3.7%), and Zn (7.7%). The relatively high carbon and nitrogen content originates from the organic HCQ moiety, while the reduced zinc percentage compared with ZnONPs further supports the incorporation of the organic ligand onto the nanoparticle surface. Elemental mapping images further indicate the simultaneous and homogeneous distribution of all four elements (C, O, N, Zn) in the HCQ-ZnONPs (Figure 8), which may demonstrate effective interaction and uniform dispersion of the organic and inorganic components.

### 3.8. FT-IR Spectra

The IR spectral data were analyzed to identify functional groups and determine the mode of complexation between the ligands and the metal ions. The FT-IR spectra for HCQ and the HCQ-ZnONPs were recorded in the range of 400–4000 cm^−1^. In HCQ, the -NH stretching vibration appears as peaks in the range of 3089–3212 cm^−1^, and the -OH frequency appears at 3417 cm^−1^ in agreement with previous reports. Following association with ZnONPs, slight shifts in the –NH and –OH stretching frequencies were observed, indicating changes in the local chemical environment of HCQ. In addition, absorption bands at 529 cm^−1^ and 584–605 cm^−1^ were assigned to Zn–O lattice vibrations and Zn–N/metal–ligand interaction-related vibrations, respectively. These spectral changes suggest an interaction between HCQ and the ZnONPs surface (Figure 9).

In the present study, the computational insights, together with our experimental observations, suggest that the single quinoline nitrogen of the HCQ molecule interacts with one exposed Zn^2+^ site on the surface of a ZnONPs cluster (containing 4 molecules of ZnONPs). The proposed interaction model is supported by complementary characterization results. The mass spectrum showed an HCQ-ZnONPs cluster ion, suggesting an interaction between HCQ and four units of ZnONPs. FT-IR spectroscopy showed shifts in the quinoline and C–N vibrational bands together with the characteristic Zn-O stretching vibration, suggesting an interaction between HCQ and ZnONPs. Likewise, the downfield shifts in the aromatic protons observed in the ^1^H NMR spectra are consistent with reduced electron density within the quinoline ring and may indicate interaction of the quinoline nitrogen with exposed surface Zn^2+^sites. XRD analysis suggested a reduction in the long-range crystallinity of ZnONPs following organic HCQ association, resulting in an amorphous nature. SEM and TEM images suggested morphological changes after HCQ association. These findings support the proposed model (Figure 1), which highlights the potential of ZnONPs as efficient carriers that enhance the physicochemical stability and biological efficacy of hydroxychloroquine [42,43].

### 3.9. Cytotoxicity Analysis

Cell viability and cytotoxicity were assessed using the resazurin dye, with results measured by UV spectrophotometry. During a 4 h incubation period, metabolically active cells converted the blue, “non-fluorescent resazurin” dye into pink, “fluorescent resorufin”. The compounds were analyzed against the positive control, and all compounds were significantly less toxic than the positive control. As the concentration of the complex decreased (10 mg/mL to 1.25 mg/mL), there was a notable reduction in cytotoxicity (% cytotoxicity). ZnONPs were found to be less toxic and more biocompatible than HCQ. The complex maintained consistent cytotoxicity across concentrations, with cytotoxicity below 20%. These ANOVA results indicate no statistically significant differences relative to the control culture, which exhibited excellent cytocompatibility (*p* < 0.05). This also indicates that, after complexation, there is no significant increase in cytotoxicity (% cytotoxicity; Figure 10A).

Figure 10B shows representative phase-contrast micrographs of cells treated with (i) HCQ, (ii) ZnONPs, (iii) HCQ-ZnONPs and controls (untreated and treated with DMSO). Compared with the untreated control (iv(a)), treated groups (i)–(iii) demonstrate varying degrees of morphological damage, reduced confluency, and loss of adherence at higher concentrations, consistent with concentration-dependent cell viability. However, it is evident from the images that DMSO-treated cells, followed by higher concentrations of HCQ and ZnONPs-treated cells, exhibit loss of normal morphology, reduced confluency, cell rounding, and detachment, indicating significant cytotoxicity and poor cell viability; whereas HCQ-ZnONPs-treated cells maintain a more intact monolayer and normal morphology. At lower concentrations, these were as confluent as untreated cells.

### 3.10. Total Antioxidant Capacity

The Total Antioxidant Capacity Assay is based on the reduction of Cu^2+^ to Cu^+^ by antioxidants present in the sample. The reduced Cu^+^ forms a colored complex with a chromogenic reagent, which is quantified spectrophotometrically at 570 nm. The antioxidant capacity is expressed as Trolox equivalents, reflecting the cumulative action of all antioxidants present in the sample. Using the BioVision TAC assay. As shown in Figure 11, the total antioxidant capacity of HCQ, ZnONPs, and the HCQ-ZnONPs complex was evaluated as Cu^2+^ reduction (µmol/µL). It showed a concentration-dependent increase in antioxidant capacity, with higher TAC values at higher concentrations (10 > 5 > 2.5 > 1.25 mg/mL).

Among the samples, ZnONPs demonstrated higher antioxidant capacity than HCQ at all concentrations, indicating the intrinsic redox potential of ZnONPs. Notably, the HCQ-ZnONPs complex consistently exhibited the highest TAC values across all tested concentrations compared with HCQ, confirming a synergistic enhancement of antioxidant activity upon complex formation. At the lowest concentration, its activity remained comparable to ZnONPs, indicating enhanced performance. At 10 mg/mL, the HCQ-ZnONPs complex achieved the maximum Cu^2+^ reduction, exceeding both HCQ and ZnONPs.

The enhanced antioxidant performance of the complex can be attributed to the combined electron-donating ability of HCQ and the high surface area-mediated redox activity of ZnONPs, which facilitates more efficient Cu^2+^ to Cu^+^ reduction. Additionally, nanoparticle conjugation improves molecular stability and accessibility of active sites, leading to superior cumulative antioxidant effects.

Overall, these findings demonstrate that nanoparticle conjugation significantly improves total antioxidant capacity, supporting the potential of HCQ-ZnONPs as an effective antioxidant system.

### 3.11. Nitric Oxide Estimation

The Griess assay indirectly estimates nitric oxide production by measuring nitrite, a stable oxidative metabolite of NO, via the formation of a pink azo dye that absorbs at 540 nm.

Nitric oxide production was quantified using the Griess reagent assay at different concentrations (1.25–10 mg/mL) of HCQ, ZnONPs, and the HCQ-ZnONPs complex. The Griess assay indirectly estimates nitric oxide production by measuring nitrite, a stable oxidative metabolite of NO, via the formation of a pink azo dye that absorbs at 540 nm. Data are expressed as mean ± SD (n = 2). Statistical analysis was performed using a two-way ANOVA, followed by appropriate post hoc multiple-comparison tests to evaluate the effects of treatment type and concentration.

The analysis revealed a statistically significant effect of concentration on NO production (*p* < 0.001), confirming a dose-dependent response for all compounds. In addition, treatment type significantly influenced NO levels (*p* < 0.01), indicating differential modulation of nitric oxide by HCQ, ZnONPs, and HCQ-ZnONPs. Importantly, a significant interaction between concentration and treatment (*p* < 0.01) was observed, indicating that the effect of concentration varied across formulations.

At higher concentrations (5 and 10 mg/mL), ZnONPs produced significantly higher NO levels compared with HCQ- ZnONPs (*p* < 0.05), while HCQ-ZnONPs consistently exhibited significantly reduced NO production relative to both HCQ and ZnONPs. At lower concentrations (1.25 and 2.5 mg/mL), although NO levels were reduced across all groups, the nanocomposite maintained lower NO levels, with differences less pronounced.

These findings validate the superior efficacy of the nanocomposite formulation compared with its individual components (Figure 12).

### 3.12. Anti-Inflammatory Activity

HCQ has a well-documented history of anti-inflammatory applications. However, increasing resistance to it necessitates the search for alternatives. To assess the anti-inflammatory efficacy of new compounds and identify better alternatives, the BSA assay was conducted in triplicate on HCQ, ZnONPs, and HCQ-ZnONPs, using celecoxib as a standard, with results showing concentration-dependent inhibition. The results indicated that HCQ-ZnONPs exhibited superior activity compared with HCQ and ZnONPs alone. The percentage inhibition (% inhibition) decreased with lower concentrations, with the complex (HCQ-ZnONPs) demonstrating a maximum % inhibition of 91% at a concentration of 5 mg/mL (Figure 13). The IC_50_ values were 3.93 μg/mLfor HCQ, 5.74 μg/mL for ZnONPs, and 1.22 μg/mL for the HCQ-ZnONPs formulation, indicating the highest inhibitory potency of the nanoformulation. This enhanced efficacy can be attributed to the strongly synergistic interaction between HCQ and ZnONPs, as indicated by a Combination Index (CI) of 0.28 at the IC_50_ level (Chou–Talalay method), together with improved nanoparticle-mediated drug delivery and greater accumulation at the target site. Significant differences among means were observed between the groups at all concentrations (*p* = 0.000177).

### 3.13. Anti-Malarial Activity

The in vitro antimalarial activity of HCQ, ZnONPs, and the HCQ-ZnONPs complex against *Plasmodium falciparum* yielded IC_50_ values of 0.82, 0.91, and 0.79 μg/mL, respectively, demonstrating that the HCQ-ZnONPs complex exhibited the greatest inhibitory activity (Figure 14). This enhanced efficacy can be attributed to the moderate synergistic interaction between HCQ and ZnONPs, as indicated by a Combination Index (CI) of 0.9 at the IC_50_ level (Chou–Talalay method), together with improved nanoparticle-mediated drug delivery and greater accumulation at the target site [47]. Additionally, ZnONPs may complement the antiplasmodial action of HCQ through reactive oxygen species generation, while HCQ inhibits heme polymerization within the parasite. Beyond direct parasite inhibition, malaria pathogenesis can also be suggested by excessive oxidative stress and inflammatory responses, including elevated reactive oxygen species, pro-inflammatory cytokines (TNF-α, IL-1β, and IL-6), and dysregulated nitric oxide production, which contribute to erythrocyte membrane damage, endothelial dysfunction, and severe disease manifestations, thereby supporting improved therapeutic outcomes [48,49,50].

### 3.14. Drug Loading and Encapsulation Efficiency

The drug loading and encapsulation efficiency of the synthesized HCQ-ZnONPs were determined by a UV-visible spectrophotometric method. HCQ was quantified at 343 nm using an HCQ calibration curve, which showed good linearity over the investigated concentration range (R^2^ = 0.9617). The amount of unassociated HCQ remaining in the supernatant was determined from the calibration equation. The average drug loading of the complex was 21.4 ± 1.80%, whereas the encapsulation efficiency was 88.48 ± 1.20%.

### 3.15. In Vitro Drug Release

The in vitro release of HCQ from the HCQ-ZnONPs complex was evaluated in PBS (pH 7.4) for 24 h using a USP dissolution method. HCQ was quantified at 343 nm using an HCQ calibration curve described by A = 0.0075C + 0.087, with good linearity (R^2^ = 0.9926) (Figure 15i). Based on the experimentally determined drug loading of 21.4%, the 30 mg HCQ-ZnONPs contained approximately 6.42 mg of HCQ. The formulation showed an initial relatively rapid release, with 19.00 ± 0.44% HCQ released at 0.5 h, followed by a gradual increase to 26.67 ± 1.33%, 32.02 ± 0.61%, 37.12 ± 1.15%, 38.73 ± 1.48%, and 44.29 ± 0.91% at 1, 2, 4, 6, and 12 h, respectively as shown in Figure 15ii. At 24 h, the cumulative HCQ release reached 48.36 ± 1.58%. The release data obtained were fitted to equations for zero-order, first-order, Higuchi and Korsmeyer–Peppas release models. Kinetic analysis showed the highest correlation with the Korsmeyer–Peppas model (R^2^ = 0.9419, n = 0.316), followed by the Higuchi model (R^2^ = 0.8731), indicating that Fickian diffusion may contribute substantially to the observed release behavior (Figure 15iii,vi). The release profile therefore demonstrated an initial faster phase followed by a more gradual release phase over the 24 h period.

## 4. Conclusions

In this study, a novel hydroxychloroquine–zinc oxide nanocluster system (HCQ-ZnONPs), integrating a bioactive organic pharmacophore with a multifunctional inorganic nanomaterial, was successfully synthesized, structurally characterized, and biologically evaluated. Comprehensive spectroscopic (^1^H NMR, FT-IR, DLS-zeta potential, and mass spectrometry), structural (XRD), and microscopic (TEM, FE-SEM, and EDAX) analyses supported the formation of the HCQ-ZnONPs hybrid system, with the proposed interaction involving the quinoline nitrogen of HCQ and exposed Zn^2+^ sites of the ZnO nanocluster. The HCQ-ZnONPs showed a drug loading of 21.4% and approximately 48.4% cumulative HCQ release over 24 h at pH 7.4, demonstrating their capacity for HCQ association and gradual release. Compared with HCQ and ZnONPs, the HCQ-ZnONPs exhibited improved cytocompatibility along with enhanced in vitro antiplasmodial and anti-inflammatory activities. The reduced IC_50_ against *Plasmodium falciparum* was consistent with a moderate synergistic antimalarial effect, whereas the anti-inflammatory response showed strong synergism. Molecular docking against PfDHFR-TS further indicated favorable interactions and provided supportive, hypothesis-generating insights that require experimental validation. In addition to antiplasmodial activity, the observed anti-inflammatory, nitric oxide scavenging, and total antioxidant capacity (TAC) responses may be relevant to malaria-associated oxidative and inflammatory processes. Collectively, these findings demonstrate the multifunctional biological properties of the HCQ-ZnONPs system and support its further investigation as a nano-enabled antiplasmodial platform. Further studies, including mechanistic investigations, pharmacokinetic evaluation, in vivo efficacy, and safety assessment, are required to establish its therapeutic potential.

## Figures and Tables

**Figure 3:**
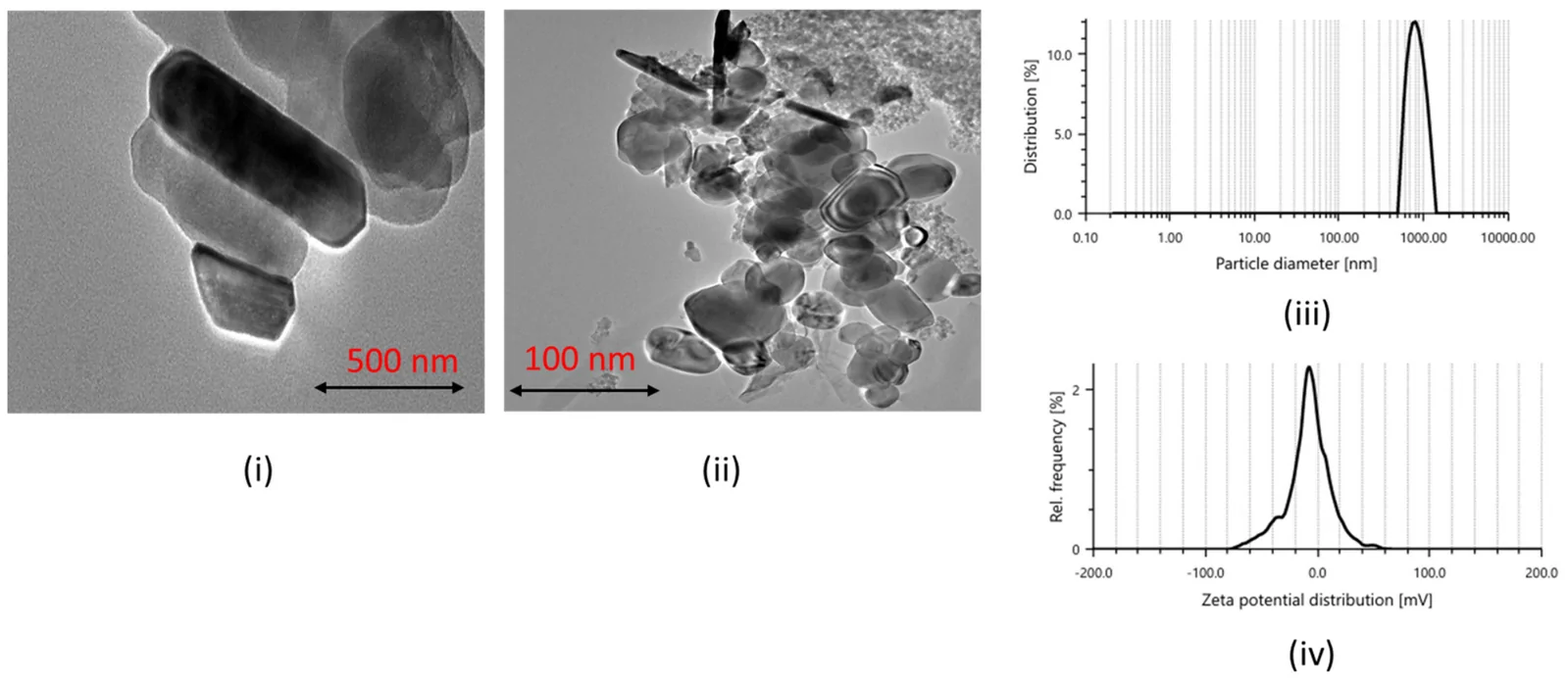
(**i**) The TEM image of ZnONPs, (**ii**) The TEM image of HCQ-ZnONPs, (**iii**) DLS analysis shows the average hydrodynamic size of HCQ-ZnONPs, (**iv**) Zeta potential of HCQ-ZnONPs.

**Figure 4:**
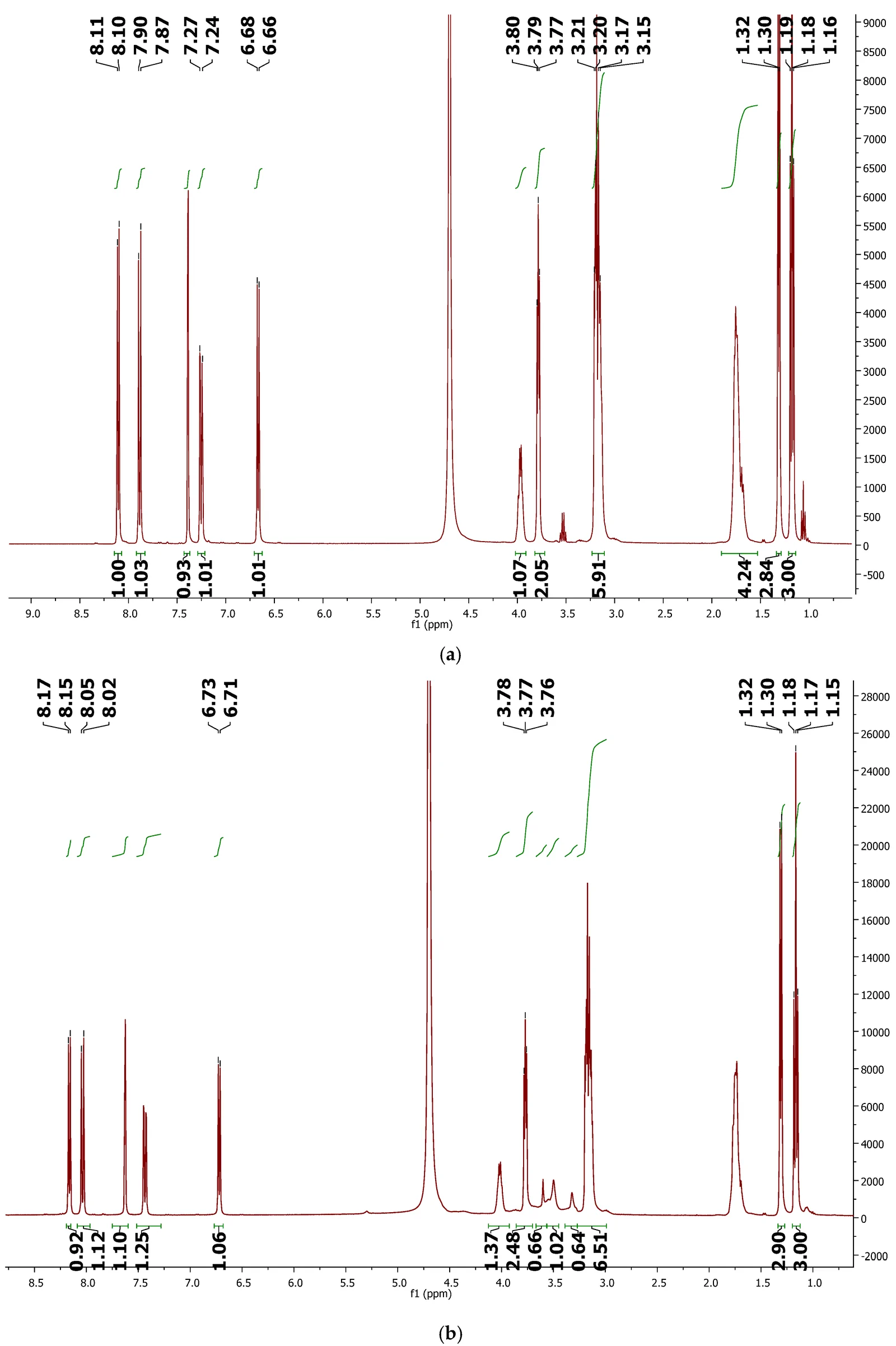
^1^HNMR spectra of (**a**) HCQ, (**b**) HCQ-ZnONPs.

**Figure 5:**
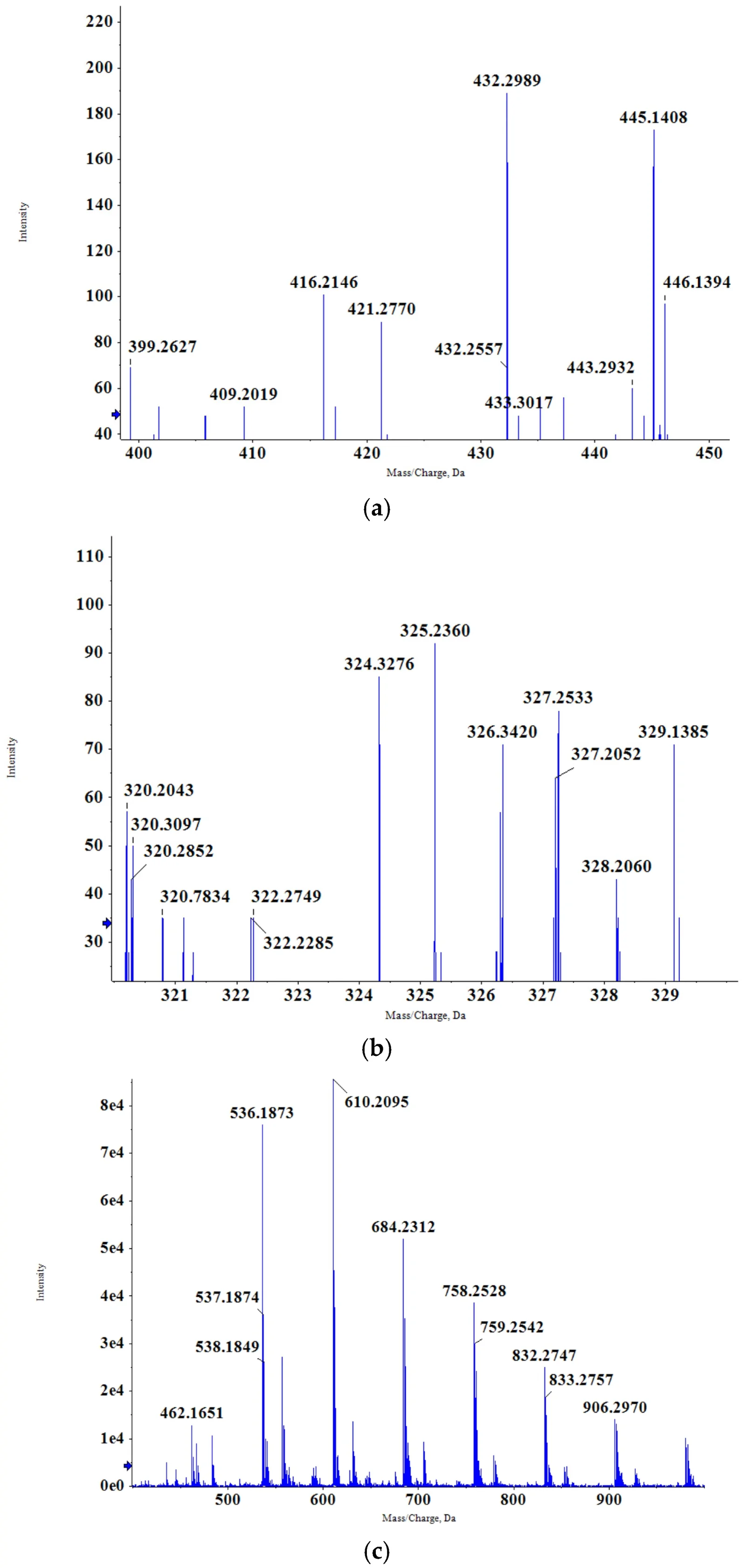
(**a**) Mass spectra of HCQ, (**b**) Mass spectra of ZnONPs, (**c**) Mass spectra of HCQ-ZnONPs.

**Figure 6:**
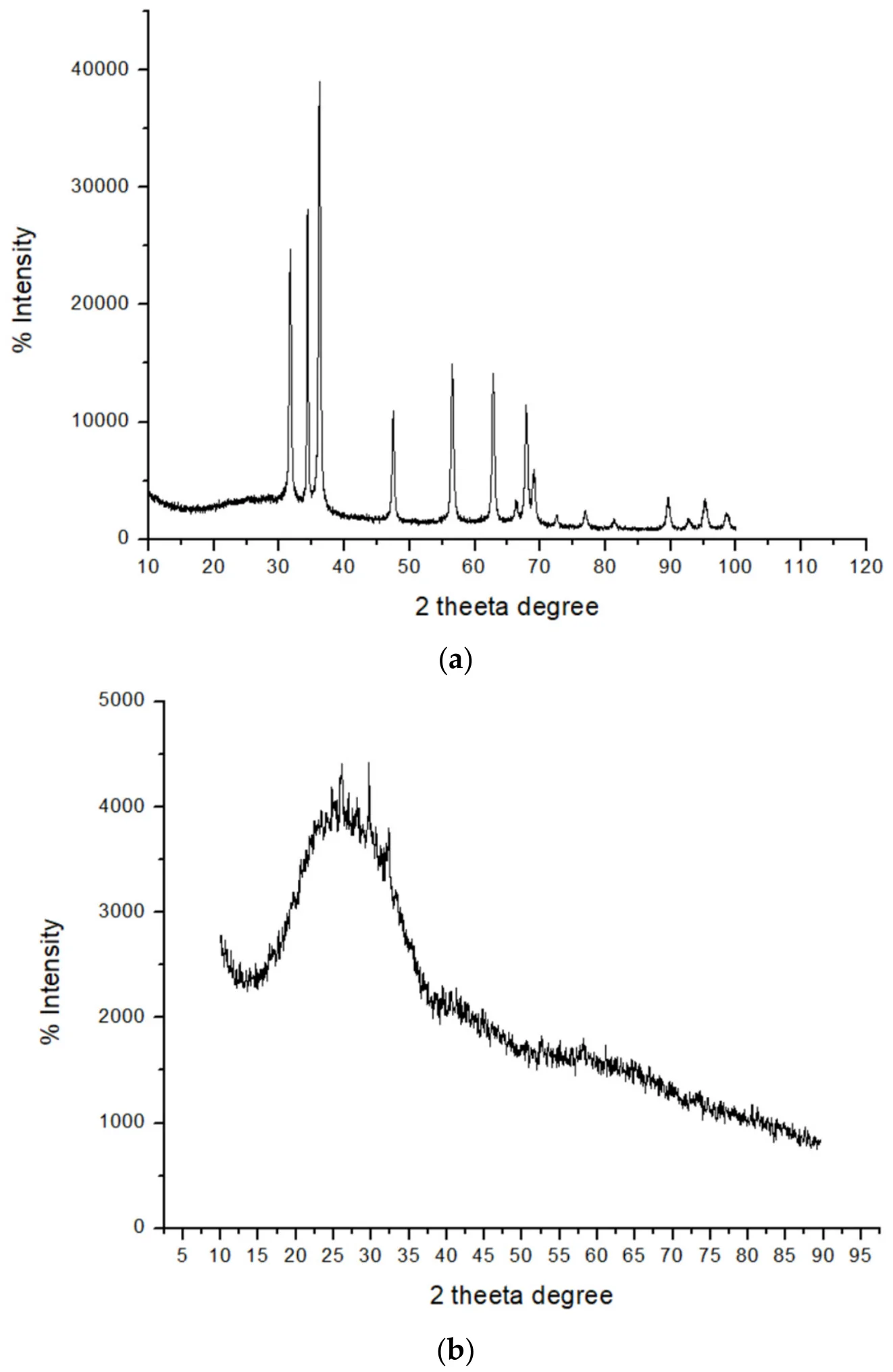
(**a**) Powder XRD pattern of ZnONPs, (**b**) HCQ-ZnONPs.

**Figure 7:**
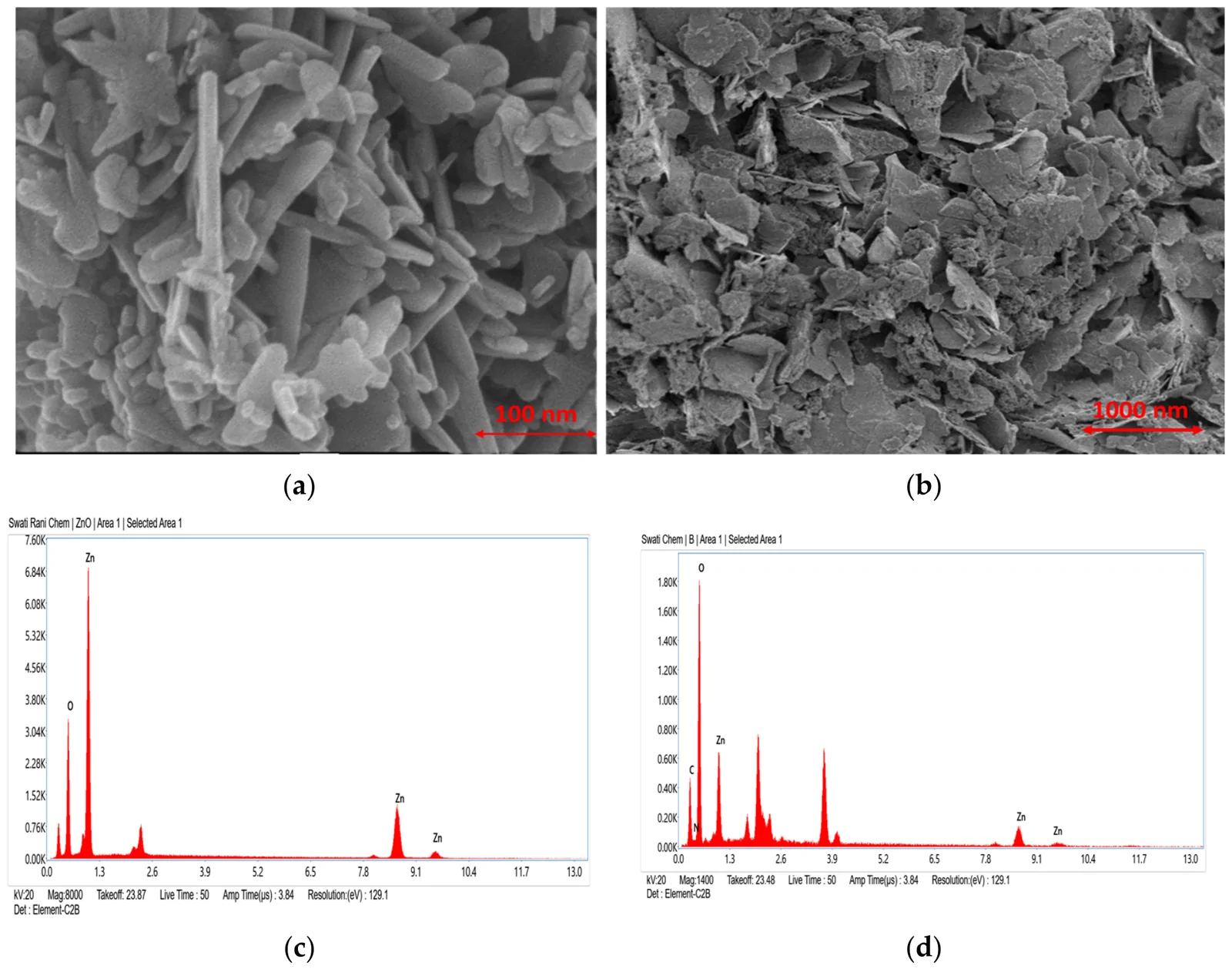
(**a**) SEM image of ZnONPs; (**b**) SEM image of HCQ-ZnONPs; (**c**) EDAX image of ZnONPs; (**d**) EDAX image of HCQ-ZnONPs.

**Figure 8:**
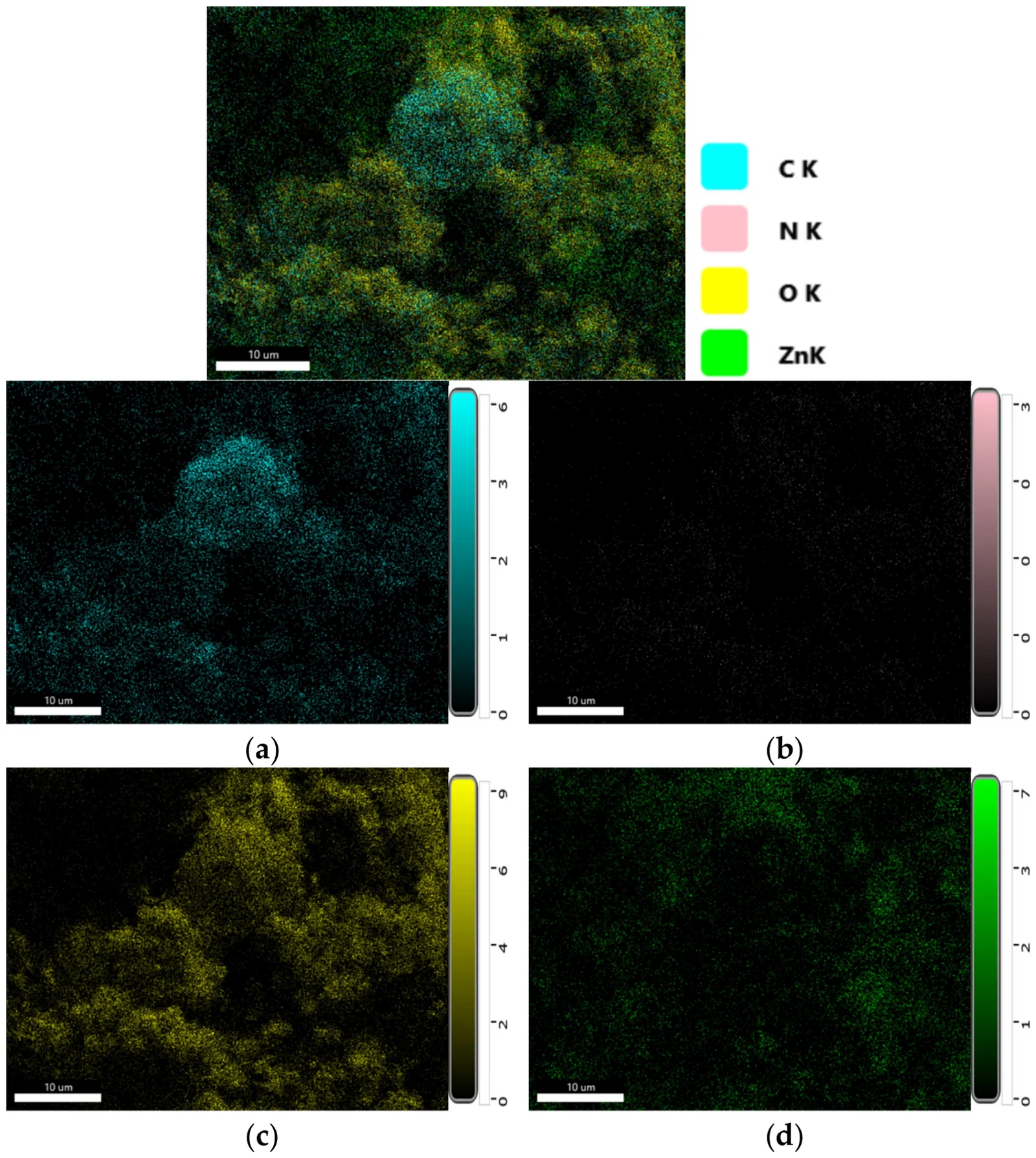
Mapping image of overlapping elements (**a**) “carbon (C)”, (**b**) “nitrogen (N)”, (**c**) “oxygen (O)”, (**d**) “zinc (Zn)” present in HCQ-ZnONPs.

**Figure 9:**
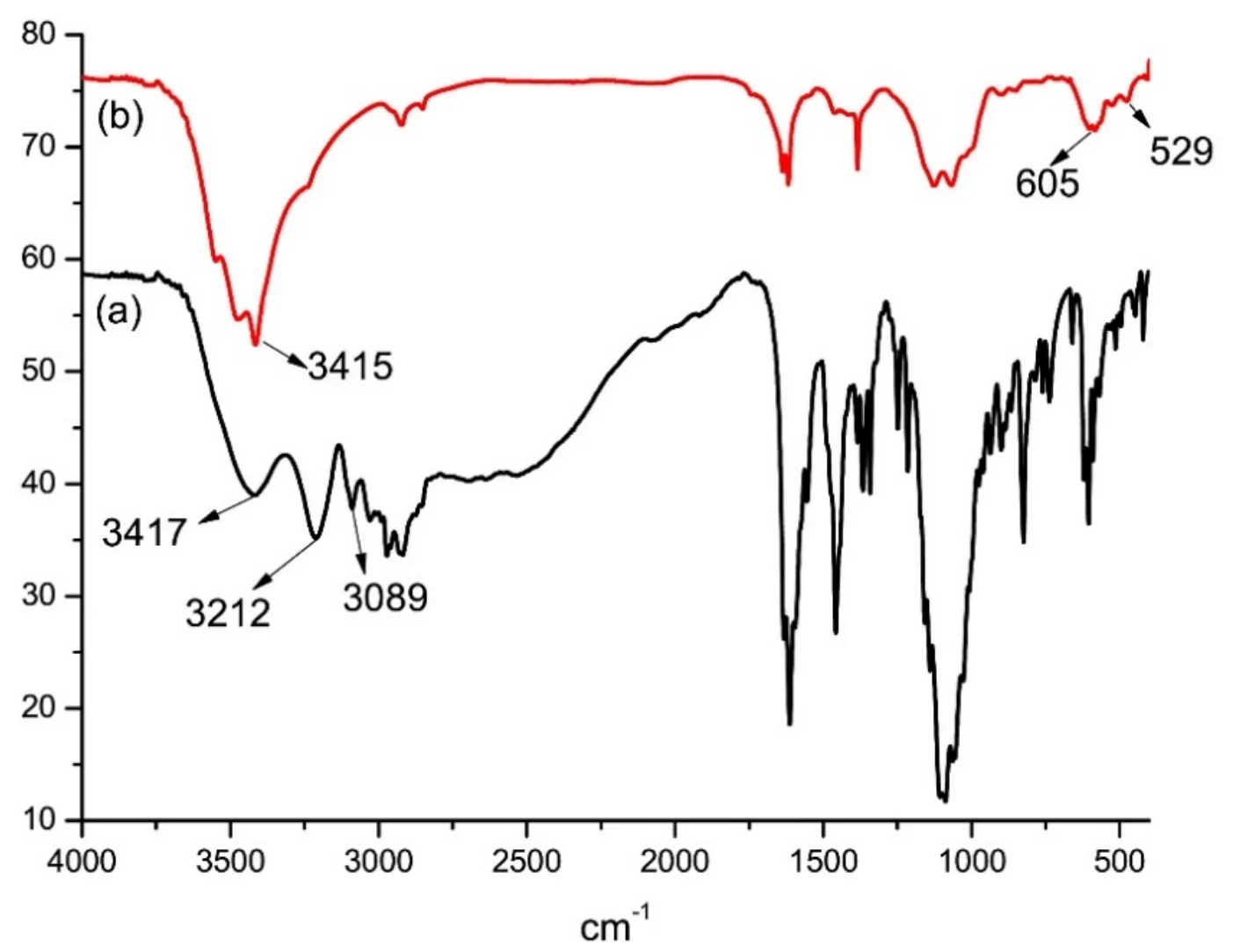
Infrared spectroscopy of (a) HCQ, (b) HCQ-ZnONPs.

**Figure 10:**
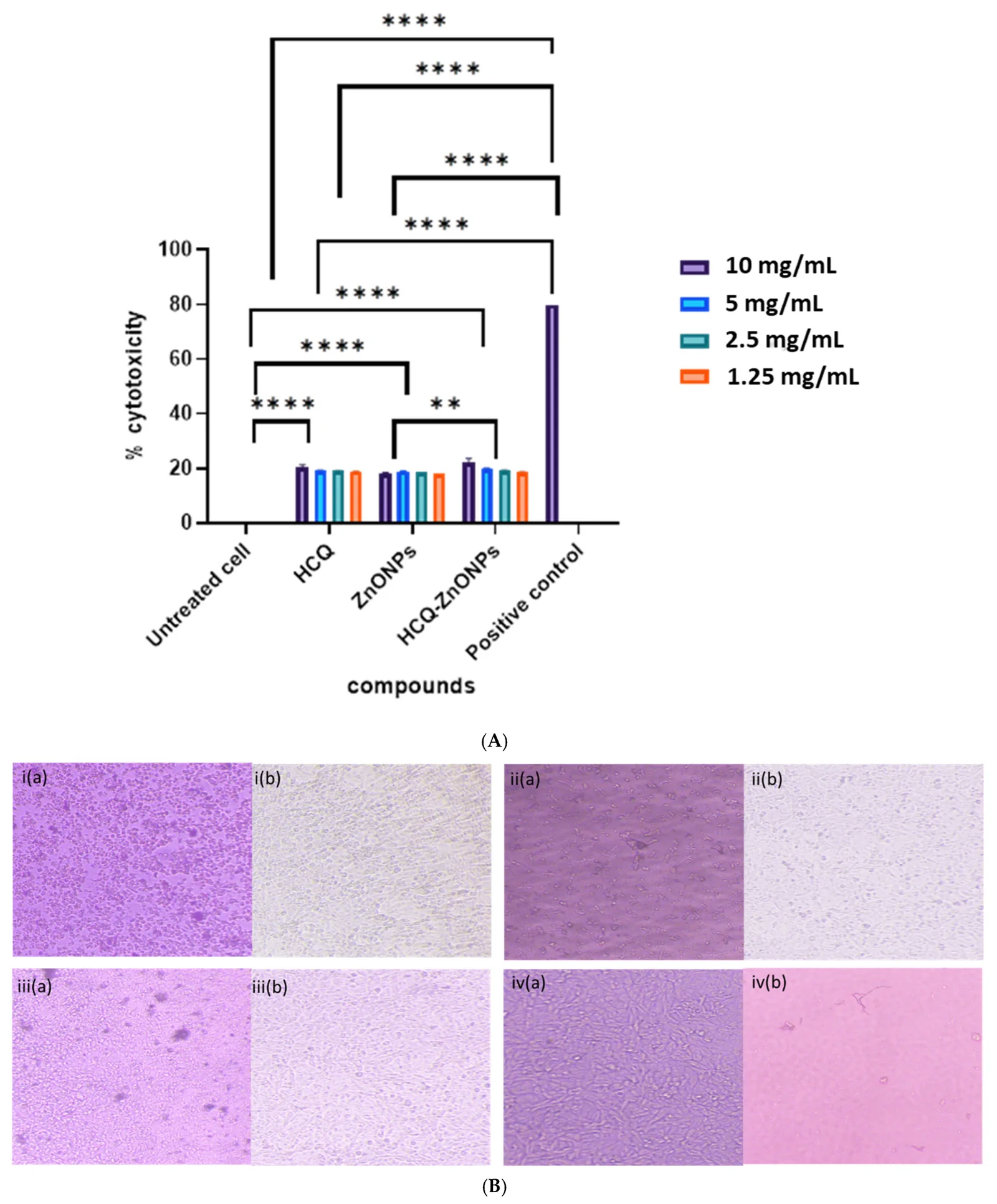
(**A**). Relative cytotoxicity of HCQ, ZnONPs and HCQ-ZnONPs-treated Vero cells at different concentrations. Asterisk indicates the level of statistical significance among the groups; ** *p* < 0.01 and **** *p* < 0.0001. (**B**). Representative phase-contrast microscopic images of Vero cells treated (**i**) HCQ ((**a**)—10 mg/mL, (**b**)—1.25 mg/mL), (**ii**) ZnONPs ((**a**)—10 mg/mL, (**b**)—1.25 mg/mL), (**iii**) HCQ-ZnONPs ((**a**)—10 mg/mL, (**b**)—1.25 mg/mL), (**iv**(**a**)) Untreated cells and (**iv**(**b**)) Cells treated with DMSO.

**Figure 11:**
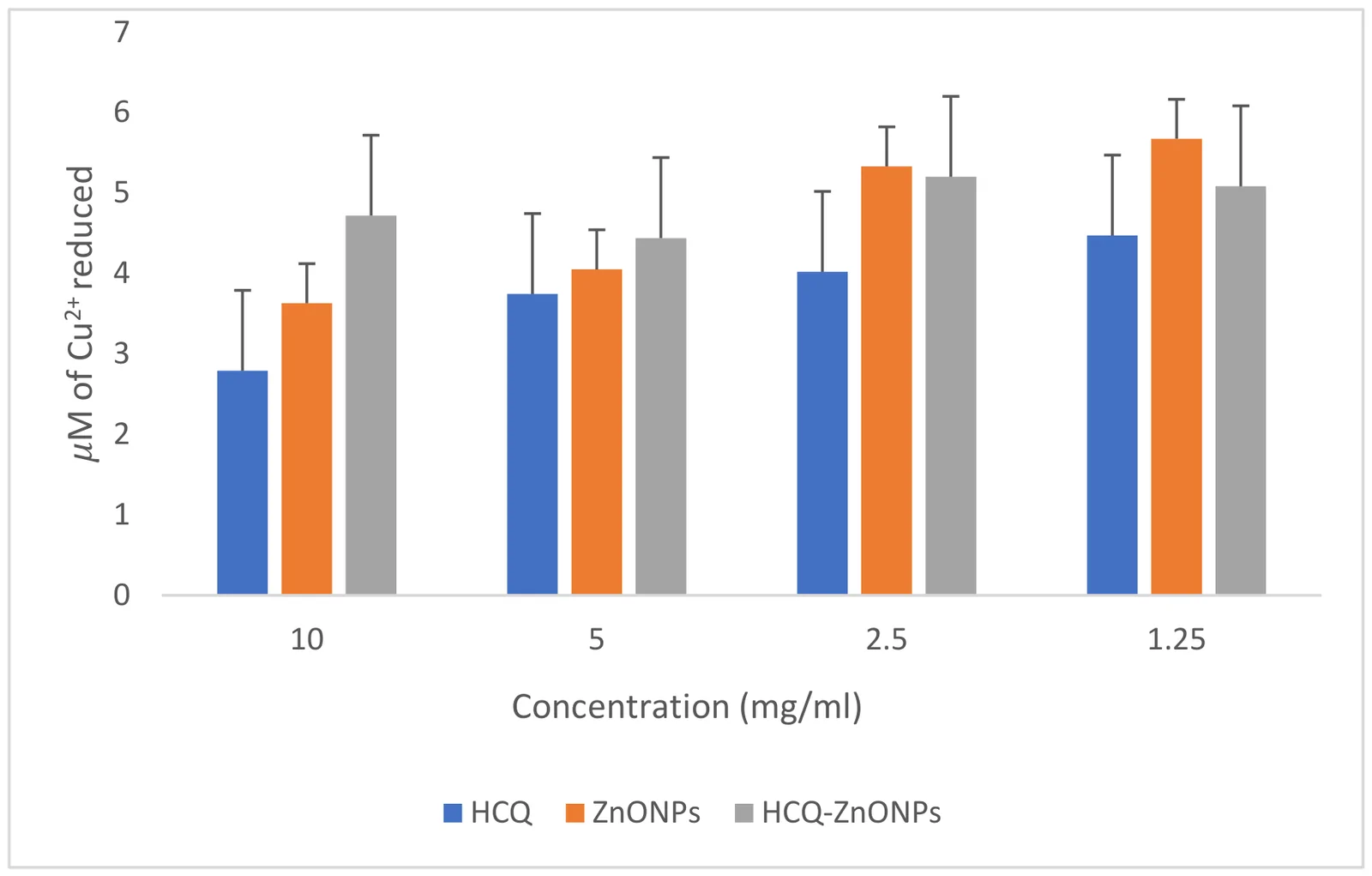
Total antioxidant capacity of HCQ, ZnONPs, and HCQ-ZnONPs at different concentrations measured by the BioVision TAC assay. Results are expressed as µmol Cu^2+^ reduced per µL.

**Figure 12:**
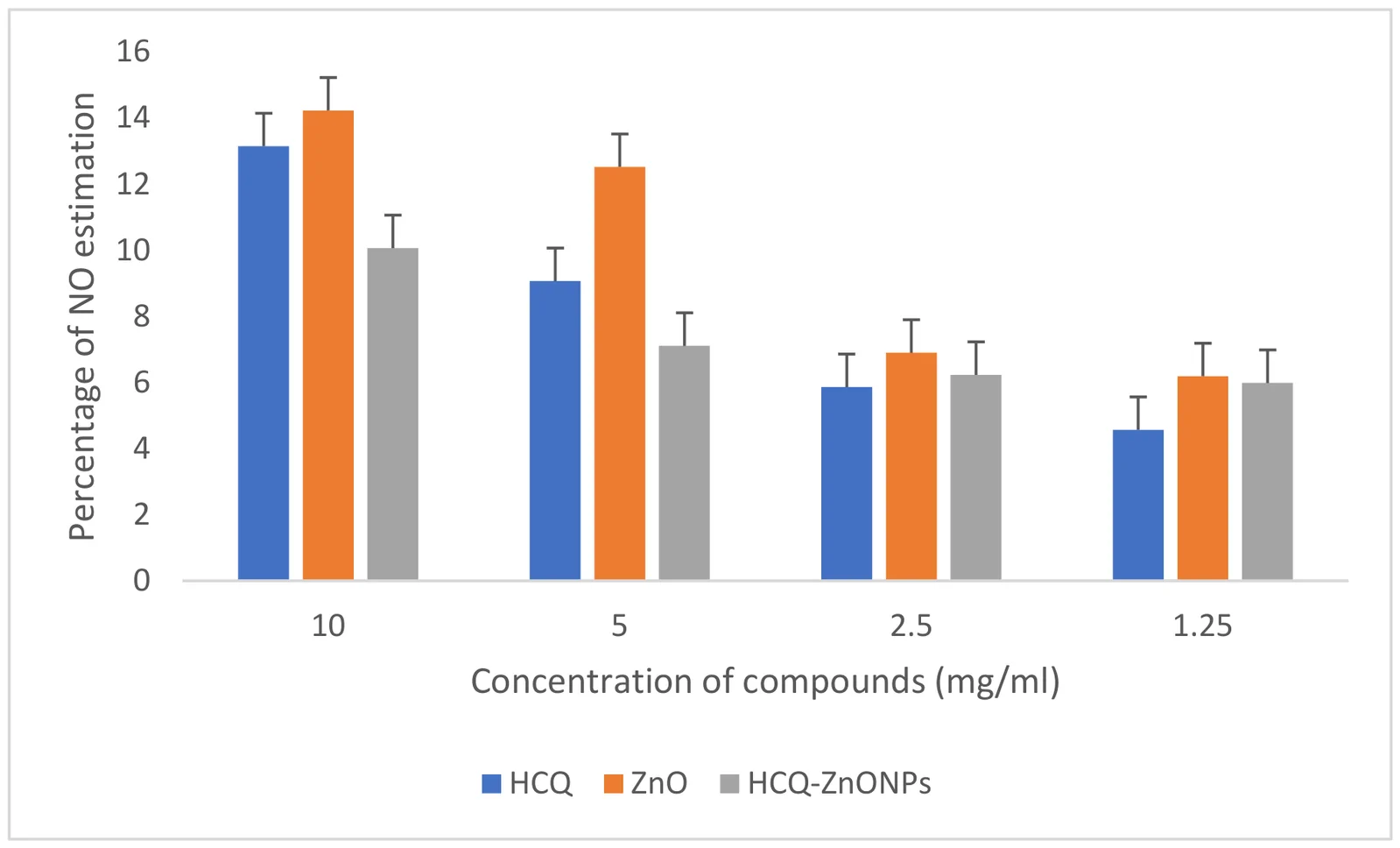
Effect of HCQ, ZnONPs, and HCQ-ZnONPs on nitric oxide (NO) production as determined by the Griess assay. Data are expressed as mean ± SD (n = 2). Statistical analysis was performed using two-way ANOVA followed by multiple comparison tests. *p* < 0.05, *p* < 0.01, and *p* < 0.001 were considered statistically significant.

**Figure 13:**
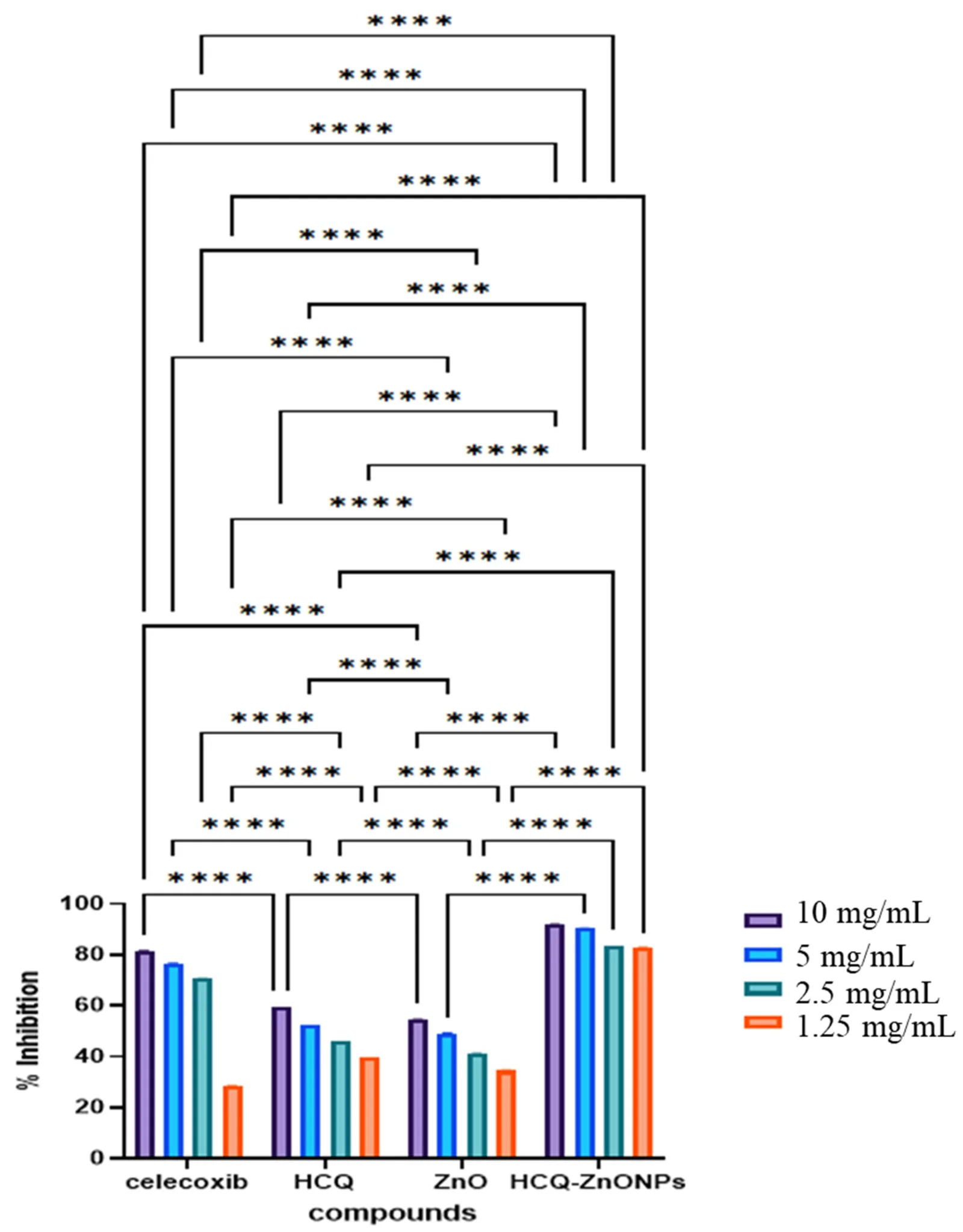
Anti-inflammatory activity of HCQ, ZnONPs, HCQ-ZnONPs, and standard celecoxib at different concentrations. Statistically different among different groups. “**** Statistically significant difference among means when compared with standard (*p* > 0.05)”.

**Figure 14:**
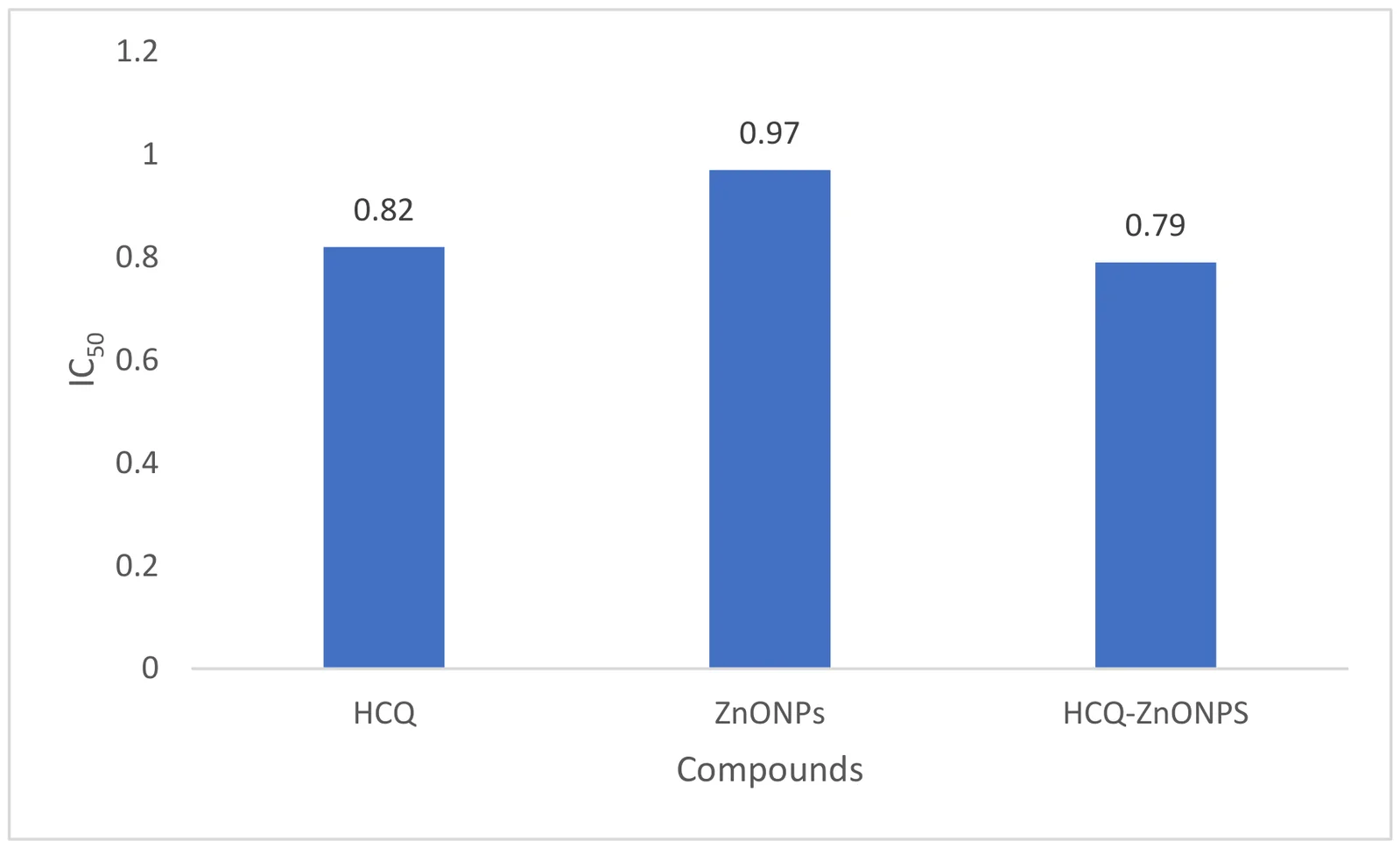
Comparative IC_50_ values of hydroxychloroquine (HCQ), zinc oxide nanoparticles (ZnONPs), HCQ-ZnONPs complex against *Plasmodium falciparum*. The graph illustrates the relative antimalarial potency of each compound, with lower IC_50_ values indicating higher activity.

**Figure 15:**
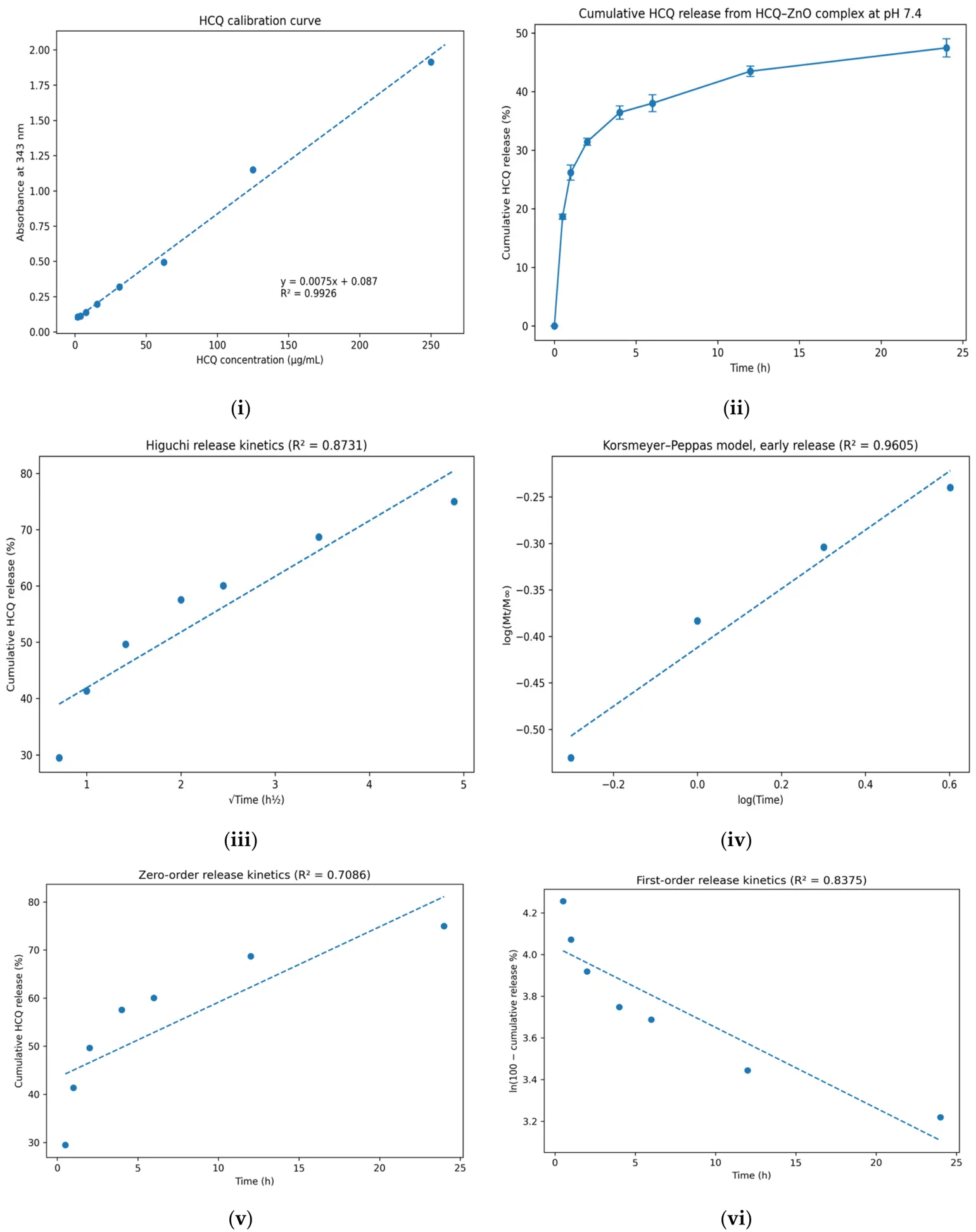
In vitro drug release and kinetic analysis of HCQ-ZnONPs at pH 7.4: (**i**) HCQ calibration curve at 343 nm; (**ii**) cumulative HCQ release profile over 24 h; (**iii**) Higuchi release kinetics; and (**iv**) Korsmeyer–Peppas release kinetics; (**v**) Zero-order kinetics; (**vi**) First-order kinetics.

## Data Availability

The authors declare that all data supporting the findings of this study are available within the article.
